# Diversification and deployment of PRR and NLR immune receptors in potato

**DOI:** 10.1111/tpj.71002

**Published:** 2026-07-01

**Authors:** Yerisf C. Torres Ascurra, Barbara Schrammel, Daniel Monino‐Lopez, Guillermo Merino Martin, Jasmin Wiedmer, Vivianne G. A. A. Vleeshouwers

**Affiliations:** ^1^ Plant Molecular and Cellular Biology Laboratory Salk Institute for Biological Studies La Jolla California 92037 USA; ^2^ Plant Breeding Wageningen University and Research 6708 PB Wageningen the Netherlands; ^3^ Graduate School Experimental Plant Sciences Wageningen the Netherlands

**Keywords:** diversification, evolution, immunity, *Phytophthora infestans*, plant receptors, potato, Solanum

## Abstract

Wild relatives of potato (*Solanum tuberosum*) have accumulated a wide diversity of immune receptors that provide disease resistance to a multitude of pathogens. The classical resistance genes in potato generally belong to the nucleotide‐binding leucine‐rich repeat (NLRs) receptors, which are well studied and widely applied in disease resistance breeding. Less explored are the pattern recognition receptors (PRRs), which include receptor‐like proteins and receptor kinases. Despite their distinct evolutionary origins and the traditional separation between PRR‐ and NLR‐mediated immune pathways, emerging molecular evidence indicates that these systems converge in their downstream signaling to combat pathogens. The identification of new PRRs in potato has revealed an unexpected diversification that promises to offer new alternatives to build resistance. By integrating mechanistic, evolutionary, and bioengineering perspectives, we outline how receptor diversity in wild *Solanum* species can be strategically harnessed to achieve durable resistance against *Phytophthora infestans* and other major potato pathogens. Elucidating the signaling networks underlying these receptors will be critical to guide their rational stacking and precise engineering, opening new opportunities to achieve durable disease resistance in potato.

## Significance Statement

Wild *Solanum* species contain a vast repertoire of immune receptors that can be harnessed to improve potato disease resistance. Emerging molecular insights reveal that pattern recognition receptors and nucleotide‐binding leucine‐rich repeat (NLR) immune receptors converge in downstream signaling, opening opportunities to strategically stack and engineer them to achieve more durable resistance to *Phytophthora infestans* and other major pathogens.

## INTRODUCTION

Plants rely on immune receptors to detect invading pathogens and pests including bacteria, fungi, oomycetes, viruses, insects, nematodes, and others. The first layer of immunity is mediated by cell‐surface pattern recognition receptors (PRRs) that respond to microbial‐ or pathogen‐associated molecular patterns (MAMPs or PAMPs) (Nurnberger & Kemmerling, 2006). The second layer is mediated by intracellular nucleotide‐binding leucine‐rich repeat (NLRs) receptors that recognize effectors delivered inside the cell (Jones & Dangl, 2006). Although PRR‐ and NLR‐mediated immune responses were initially considered largely independent layers of plant defense, the concept of mutual potentiation of immunity by PRRs and NLRs, and the strong correlation between the number of genes encoding these two types of receptors suggests that both receptors cooperate and may evolve simultaneously to provide a robust defense response (Gong et al., 2022; Ngou et al., 2021; Pruitt et al., 2021). Moreover, PRR and NLR signaling pathways converge and are interconnected (Yuan, Jiang, et al., 2021), supporting the view of a unified immune system. However, supporting evidence for this currently evolving model is derived mainly from studies in Arabidopsis and remains to be validated in other species.

Potato (*Solanum tuberosum*) is a staple crop underpinning global food security, yet its cultivation is threatened by a multitude of devastating diseases, most notably late blight caused by the oomycete *Phytophthora infestans* (Haas et al., 2009; Wang et al., 2026). Fortunately, abundant resistance sources occur in wild *Solanum* species, particularly among tuber‐bearing relatives from the highly diverse section *Petota*, distributed across North, Central, and South America (Spooner et al., 2004; Vleeshouwers, Finkers, et al., 2011; Vleeshouwers & Jacobsen, 2024). Interestingly, *Solanum* genomes exhibit striking expansions of their NLRs, with an exceptionally large array of approximately 400 to more than 800 NLRs (Jupe et al., 2013; Potato Genome Sequencing Consortium, 2011; Tang et al., 2022). This genetic reservoir, alongside related non‐tuber‐bearing species like *Solanum americanum* (Lin et al., 2023; Witek et al., 2021), has been forged through a continuous evolutionary arms race with local pathogens. Consequently, these wild relatives encode an extensive repertoire of innate resistance that offers a powerful, modular toolkit to combat diseases.

Traditionally, breeding for late blight resistance in potato has relied on *R* (*NLR*) genes from wild *Solanum* species. However, most NLRs have been defeated due to the fast evolution of the matching effectors of *P. infestans*, proving that *NLR* genes alone are not likely to provide durable resistance to *P. infestans* (Fry, 2008). Therefore, more natural sources and alternative strategies need to be exploited to achieve more durable and broad‐spectrum resistance in potato breeding. PRRs have been underused in potato breeding, maybe because of their relatively modest resistance phenotype compared to NLRs. However, PRRs recognize highly conserved PAMPs (Jones & Dangl, 2006), which can lead to broader and more durable defense responses. Furthermore, recent advances in receptor engineering and transfer across species make them a promising strategy to improve disease resistance in potato and other crops.

In this focused review, we argue that, similarly to findings in model plants, immune receptor evolution in *Solanum* represents a continuum rather than a dichotomy between PRR‐ and NLR‐mediated immunity. We discuss the diversity and function of the many identified NLR immune receptors across the tuber‐bearing *Solanum* section *Petota* and the non‐tuber‐bearing *S. americanum*, using *P. infestans* as a primary model. As PRRs have been understudied in potato, we include some PRRs from tomato species consistent with the evolutionary relationship. By integrating mechanistic, evolutionary, and bioengineering perspectives, we highlight how receptor diversity in wild *Solanum* species can be strategically harnessed to achieve durable resistance against *P. infestans* and other major pathogens (Box 1).

## PRRs—FIRST LINE OF DETECTION

PRRs can be classified as receptor‐like proteins (RLPs) or receptor kinases (RKs), which are distinguished by the absence or presence of an intracellular kinase domain, respectively (Couto & Zipfel, 2016). Both RLPs and RKs recognize a wide range of ligands derived from pests and pathogens using different kinds of extracellular domains including leucine‐rich repeat (LRR), lectin, malectin, lysin motif (LysM), and epidermal growth factor‐like domains (Couto & Zipfel, 2016). LRR–RKs constitute the largest RK subfamily and are the best characterized receptor‐like kinases in plants. LRR–RKs subgroup XII is specifically associated with PAMPs, DAMPs, or apoplastic effector recognition (Dufayard et al., 2017), and interestingly, the gene density varies markedly among species and shows a higher expansion compared to other subgroups (Ngou et al., 2022). Similarly, LRR–RLPs constitute the largest RLP subfamily in plants, and their gene numbers vary considerably among species (Ngou et al., 2022). This underscores the dynamic evolution of these two types of PRRs and their role in enabling plant adaptation to diverse pathogens.

**Figure 1:**
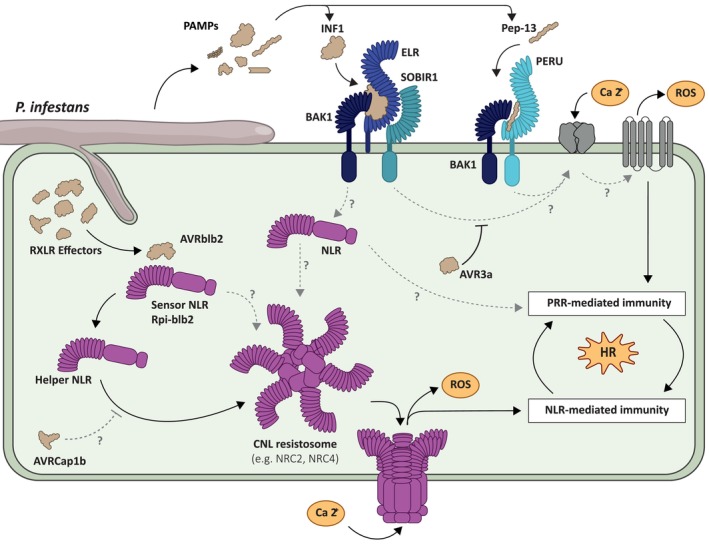
The two‐tiered plant immune system, shown for the potato—*Phytophthora infestans* interaction. Cell surface pattern recognition receptors (PRRs) such as ELR and PERU perceive the pathogen‐associated molecular patterns (PAMPs) INF1 and Pep‐13, respectively. Upon ligand binding, PERU and ELR (that constitutively interacts with SOBIR1) recruit BAK1 to initiate PRR‐mediated defense responses that include reactive oxygen species (ROS) production and hypersensitive response (HR). However, *P. infestans* secretes effectors like AVR3a to suppress these responses. In turn, potato counterattacks this suppression by intracellular nucleotide‐binding leucine‐rich repeat (NLR) receptors, like the sensor Rpi‐blb2 which detects AVRblb2, activating a robust defense response and providing resistance. *Phytophthora infestans* also secretes effectors like AVRcap1b to suppress immune activation by targeting helper NLR proteins. Dotted lines indicate interactions or signaling events for which experimental direct evidence is lacking in potato.

When PRRs recognize their corresponding ligand, they undergo dimerization or form heteromeric complexes with other co‐receptors. RKs recruit LRR‐RK BAK1/SERK3 (BRASSINOSTEROID INSENSITIVE 1‐ASSOCIATED KINASE1/SOMATIC EMBRYOGENESIS RECEPTOR KINASE 3) or CERK1 (Chitin Elicitor RK 1) as co‐receptors, while LRR‐RLPs constitutively interact with SOBIR1 (SUPPRESSOR OF BIR1‐1) and recruit BAK1 upon ligand binding (Couto & Zipfel, 2016; Dodds et al., 2024; Yang, Steidele, Huang, et al., 2025) (Figure 1). The complex formation initiates a cascade of downstream signaling events including reactive oxygen species (ROS) burst, ion fluxes, activation of mitogen‐activated protein kinase (MAPK) pathways, callose deposition, and transcriptional reprogramming that ultimately culminate in PRR‐mediated immune responses (Defalco & Zipfel, 2021). For a comprehensive overview of PRR‐related perception, complex formation, signaling networks, and evolution, readers are referred to the recent review of Ngou et al. (2026b).

*Solanum tuberosum* harbors many cell surface immune receptors. A large‐scale analysis indicated that the potato genome encodes 68 LRR‐RK‐XII and 122 LRR‐RLPs, which represent a high percentage of PRR genes compared with other species such as *Arabidopsis thaliana* (12 and 54), *Nicotiana benthamiana* (39 and 56), or *Solanum lycopersicum* (46 and 48) (Ngou et al., 2022). Despite the presence of a high number of PRRs in *S. tuberosum*, likely as a result of pathogen pressure, our understanding of their functions remains limited.

Homologs of previously identified PRRs and regulatory elements have been studied in *S. tuberosum*. The LysM‐RK StCERK1 was identified as an active kinase essential in chitin‐triggered signaling in potato, contributing to resistance against *P. infestans*, *Alternaria solani*, and *Ralstonia solanacearum* (Cao et al., 2025). FLS2 homologs are widely present in Solanaceous species, yet none have been functionally characterized in potato (Torres Ascurra et al., 2023). In *S. tuberosum*, an FLS2 homolog (StFLS2) was used to explore the regulatory element BINDING PARTNER OF ACD11 1 (StBPA1), which constitutively associates with StFLS2, StSOBIR1, and StBAK1 to negatively regulate immune responses. Upon infection, StBAK1 is recruited and phosphorylates StBPA1 to counteract the suppression and ensure a proper immune response against bacteria and oomycetes (Li, Ma, et al., 2025).

In the tuber‐bearing *Solanum* section *Petota*, two novel PRRs have been cloned and functionally characterized (Table 1). The LRR‐RLP ELICITIN RECEPTOR (ELR) was cloned from the wild potato *Solanum microdontum* (Du et al., 2015). ELR forms a complex with BAK1/SERK3 and SOBIR1 to specifically recognize diverse elicitins from *Phytophthora* species (Domazakis et al., 2020; Du et al., 2015). Transfer of ELR into cultivated potatoes resulted in enhanced resistance to *P. infestans* (Du et al., 2015). The second PRR LRR‐RK PEP‐13 RECEPTOR UNIT (PERU) was originally cloned from *S. tuberosum* group Phureja and later also from other *Solanum* species (Table 1). PERU recognizes Pep‐13 and Pep‐25, which are conserved peptide epitopes originating from cell wall glycoprotein GP42 with transglutaminase activity found in *Phytophthora* species (Torres Ascurra et al., 2023). Upon binding of Pep‐13/Pep‐25, PERU forms a complex with BAK1/SERK3 to activate immune responses like ROS burst, ethylene accumulation, and ultimately enhance the resistance to *P. infestans* (Torres Ascurra et al., 2023).

**Table 1:** Cloned and functionally characterized pattern recognition receptors (PRRs) and nucleotide‐binding leucine‐rich repeats (NLRs) from wild tuber‐bearing *Solanum* species and ancestral *Solanum americanum*

Receptor family (type)	Cloned from	Chromosome	Species origin	AVR/PAMP family	Pathogen	References
PRR						
CERK1 (RK)	*S. tuberosum*	Chr. 7	North, Central and South America	chitin	*P. infestans*, *A. solani*, *R. solanacearum*	Cao et al. (2025)
ELR (RLP)	*S. chacoense*, *S. edinense*, *S. microdontum*, *S. microd. gigantophyllum*, *S. papita*, *S. phureja*, *S. piurae*	Chr. 12	North, Central and South America	INF1	*P. infestans*	Du et al. (2015)
FLS2 (RK)	*S. tuberosum*	Chr. 2	North, Central and South America	flg22	Bacteria	Li, Jarquin Bolaños, et al. (2025)
PERU (RK)	*S. berthaultii*, *S. boliviense*, *S. doddsii*, *S. hondelmannii*, *S. leptophyes*, *S. microdontum*, *S. microd. gigantophyllum*, *S. phureja*, *S. tuberosum*	Chr. 3	North, Central and South America	Pep‐25	*P. infestans*	Torres Ascurra et al. (2023)
Ve1 (RLP)	*S. tuberosum*	Chr. 9	North, Central and South America	Ave1	*V. dahliae*, *V. alboatrum*	Song et al. (2017)
NLR						
R1 (CNL)	*S. demissum* *S. burkartii*	Chr. 5	Mexico	AVR1, AVRbrk1	*P. infestans*	Ballvora et al. (2002) Wang et al. (2026)
R2 (CNL)	*S. demissum*, *S. bulbocastanum*, *S. schenckii*, *S. hjertingii*, *S. edinense*	Chr. 4	Mexico	AVR2	*P. infestans*	Lokossou et al. (2009), Champouret (2010)
Rpi‐mcq1 (CNL)	*S. mochiquense* *S. huancabambense*	Chr. 9	Peru	AVR2	*P. infestans*	Jones et al. (2014), Aguilera‐Galvez et al. (2018), Aguilera‐Galvez (2019)
R3a (CNL)	*S. demissum*, *S. stoloniferum*	Chr. 11	Mexico	AVR3a	*P. infestans*	Huang et al. (2005), Champouret (2010)
R3b (CNL)	*S. demissum*	Chr. 11	Mexico	AVR3b	*P. infestans*	Li et al. (2011)
R8 (CNL)	*S. demissum*	Chr. 9	Mexico	AVR8	*P. infestans*	Vossen et al. (2016)
R9a (CNL)	*S. demissum*	Chr. 9	Mexico	AVR9	*P. infestans*	Kwang‐Ryong (2013)
Rpi‐blb1 (CNL)	*S. bulbocastanum*, *S. papita*, *S. stoloniferum*	Chr. 8	Mexico	AVRblb1	*P. infestans*	Van Der Vossen et al. (2003), Song et al. (2003), Vleeshouwers et al. (2008)
Rpi‐blb2 (CNL)	*S. bulbocastanum*	Chr. 6	Mexico	AVRblb2	*P. infestans*	Van Der Vossen et al. (2005)
Rpi‐chc1 (CNL)	*S. chacoense* *S. berthaultii* *S. tarijense*	Chr. 10	Bolivia	AVRchc1	*P. infestans*	Monino‐Lopez et al. (2021)
Rpi‐vnt1 (CNL)	*S. venturii*	Chr. 9	Argentina	AVRvnt1	*P. infestans*	Foster et al. (2009)
Rpi‐amr1 (CNL)	*S. americanum*	Chr. 11	Global	AVRamr1	*P. infestans*	Witek et al. (2021)
Rpi‐amr3 (CNL)	*S. americanum*, *S. cardiophylum*	Chr. 4	Global, Mexico	AVRamr3	*P. infestans*	Witek et al. (2016), Wang et al. (2026)
Rpi‐amr4 (CNL)	*S. americanum*	Chr. 1	Global	AVRamr4	*P. infestans*	Lin et al. (2023)
Rpi‐amr5 (CNL)	*S. americanum*	Chr. 12	Global	AVRamr5	*P. infestans*	Heal et al. (2025)
Rpi‐cjm1 (TNL)	*S. cajamarquense*	Chr. 1	Peru	AVRblb2	*P. infestans*	Wang et al. (2026)
Gro1‐4 (TNL)	*S. spegazzinii*	Chr. 7	Argentina	Unknown	*Globodera rostochiensis*	Paal et al. (2004)
Ry‐chc (TNL)	*S. chacoense*	Chr. 9	Bolivia	CP	Potato virus Y (PVY)	Li et al. (2022)
Ry‐sto (TNL)	*S. stoloniferum*	Chr. 12	Mexico	CP	Potato virus Y (PVY)	Grech‐Baran et al. (2019)
Y‐1 (TNL)	*S. andigena*	Chr. 11	South America	Unknown	Potato virus Y (PVY)	Vidal et al. (2002)
Hero (CNL)	*S. microdontum*	Chr. 4	Mexico	Unknown	*Globodera rostochiensis*	Ernst et al. (2002)
Gpa2 (CNL)	*S. spegazzinii*	Chr. 12	Argentina	AVRGpa2	*Globodera pallida*	Van Der Vossen et al. (2000)
Rx (CNL)	*S. acaule*	Chr. 12	North, Central and South America	CP	Potato Virus X (PVX)	Bendahmane et al. (1999)

*Petota* species originated from an ancient interspecific hybridization between *Etuberosum* and tomato species (Zhang, Zhang, et al., 2025) and consistent with this evolutionary relationship, PRRs identified in tomato provide valuable insights that can inform the discovery and functional characterization of similar receptors in potato. In *S. lycopersicum*, the LRR‐RLP SlCf‐9 was the first PRR discovered and showed to confer resistance to strains of the fungus *Cladosporium fulvum* secreting the matching apoplastic effector AVR9 (Jones et al., 1994). Subsequently, additional SlCfs including SlCf‐2, SlCf‐4, SlCf‐5, and SlHcr9‐4E were characterized (Dixon et al., 1996, 1998; Thomas et al., 1997; Westerink et al., 2004). The LRR‐RLPs LeEIX were identified in *S. lycopersicum*, as capable of binding the potent elicitor ethylene‐inducible xylanase (EIX) produced by *Trichoderma* fungi (Ron & Avni, 2004). The LRR‐RLP Ve1 from tomato detects the *Verticillium* effector Ave1, providing resistance against race 1 strains of *V. dahliae* and *V. alboatrum* (De Jonge et al., 2012; Kawchuk et al., 2001), and *Ve1* homologs were detected in potato species as well (Song et al., 2017) (Table 1). Interestingly, Ve1‐mediated resistance can be activated by Ave1 homologs from *Fusarium oxysporum* and *Cercospora beticola* (De Jonge et al., 2012). SlI‐3 was isolated from *S. pennellii* and recognizes the *F. oxysporum* f. sp. *lycopersicie* effector AVR3 to confer resistance to this pathogen (Catanzariti et al., 2015). Unlike the previous LRR‐type PRRs, SlI‐3 contains a different extracellular domain called S‐domain that includes the subdomains G‐type lectin and PAN_AP domain, conserved cysteine residues, and the presence of a PTDT‐motif (Catanzariti et al., 2015). Also, in potato, putative G‐LecRK are thought to be involved in the response to small cysteine‐rich proteins of *P. infestans* (Lin et al., 2020).

Additionally, PRRs recognizing ligands with clearer hallmarks of PAMPs have been identified in tomato as well. The LRR‐RK FLAGELLIN SENSING 3 (FLS3) binds flgII‐28, another flagellin fragment unrelated to flg22 perceived by FLS2, and enhances immune responses to bacterial infection (Hind et al., 2016). The LRR‐RK CORE (COLD SHOCK PROTEIN RECEPTOR) recognizes the peptide csp22 derived from the conserved RNP‐1 nucleic acid‐binding motif of bacterial cold‐shock proteins (CSPs) with high affinity and specificity, and when transferred to Arabidopsis enhances resistance to infection by *Pseudomonas syringae* pv. *tomato* DC3000 (Wang et al., 2016). The LRR‐RLP CUSCUTA RECEPTOR 1 (CuRe1) recognizes the peptide epitope Crip21 derived from a glycine‐rich cell wall protein (GRP) from *Cuscuta reflexa* to activate immune responses against this parasitic plant (Hegenauer et al., 2016, 2020). The higher number of characterized PRRs in tomato suggests that potato has the potential to bear PRRs that could be deployed to enhance resistance against pathogens.

### PRR‐mediated priming of NLRs signaling

Studies in Arabidopsis support a model in which PRRs and NLRs act synergistically to activate more robust immune responses and suggest that PRR signaling enhances NLR‐mediated responses which in turn promote the accumulation of components involved in PRR‐mediated immunity (Ngou et al., 2021; Pruitt et al., 2021; Tian et al., 2021; Yuan, Jiang, et al., 2021). However, to date, little is known in *Solanum* species, as this subject has mainly been explored in model systems. It has been shown that PRR co‐receptors AtBAK1/SERK3 and AtBKK1/SERK4 are required for responses mediated by Toll/interleukine‐1 receptor‐NLR (TNLs) AtRPP2 and AtRPP4 (Roux et al., 2011). In addition, immune responses mediated by the TNL pair AtRRS1‐RPS4 are compromised without prior or simultaneous activation of PRR signaling (Ngou et al., 2021). Using Arabidopsis lines encoding inducible effectors, it was shown that NLR activation alone was insufficient to trigger the hypersensitive cell death response (HR), a response mainly associated with NLRs. Strong HR occurred only when both NLR and PRR signaling were co‐activated, highlighting that PRRs potentiate NLR‐induced HR (Ngou et al., 2021). Furthermore, the activation of TNLs (AtRRS1‐RPS4, AtRPP4) and Coiled‐coil CC NLRs (CNLs) (AtRPS2, AtRPM1, AtRPS5) significantly increases the protein abundance of key PRR signaling components including BAK1, SOBIR1, BIK1, RBOHD, and MPK3 (Ngou et al., 2021; Yuan, Jiang, et al., 2021). Collectively, these findings support a model in which PRR constitutes the primary source of resistance, while NLRs potentiate and restore PRR signaling. This reinforcement involves modulation of protein turnover as well as transcriptional and translational reprogramming, resulting in increased accumulation of PRR signaling components. This overabundance may further help to outcompete suppressing effectors which target these proteins.

Whether this Arabidopsis‐derived model of mutual potentiation is fully conserved in Solanaceae is a critical question for crop improvement. In potato, our understanding is currently constrained by the limited characterization of its PRR repertoire. However, specific examples suggest a potential divergence in the intensity of the initial response. Unlike canonical PRRs described in Arabidopsis, two of the PRRs identified in potato (ELR and PERU) trigger HR upon detection of their respective ligands. For example, HR triggered by ELR upon detection of INF1 is suppressed by various RXLR effectors including AVR3a (Bos et al., 2006; Oh et al., 2009). It remains unknown if ELR and R3a potentiate each other or whether their signaling pathways show more points of interconnection. Similarly, HR triggered by PERU upon detection of Pep‐13 is expected to be suppressed by several RXLR effectors from *P. infestans*. Elucidation of these interactions would be useful to guide new breeding strategies.

### PRR transfer and engineering

PRRs intrinsic nature to recognize conserved and essential microbial signatures makes them especially valuable for engineering resistance. Accordingly, PRR transfer represents a powerful strategy to engineer broad spectrum and durable disease resistance, since PAMPs can differ in the breath of their recognition and distribution (Thomma et al., 2011). While bacterial flagellin (flg22) is recognized in mono‐ and dicotyledonous species, elongation factor Tu (elf18) recognition is restricted to Brassicaceae species (Zipfel et al., 2006). The EF‐Tu receptor EFR showcased the success of the interfamily transfer (Lacombe et al., 2010). Transgenic expression of EFR from Arabidopsis confers broad‐spectrum bacterial resistance in the Solanaceae species *S. lycopersicum* and *N. benthamiana* (Lacombe et al., 2010), and to *Ralstonia solanacearum* in *S. tuberosum* (Boschi et al., 2017). A similar case is reported for Nep1‐like proteins (nlp20) that are widely distributed in bacteria, fungi and oomycetes (Oome & Van Den Ackerveken, 2014). Transgenic expression of the Arabidopsis nlp20 receptor, RLP23, in *S. tuberosum* enhances defense responses to the major oomycete and fungal pathogens *P. infestans* and *Sclerotinia sclerotiorum* (Albert et al., 2015). Interestingly, Pep‐13 was found widely present in *Phytophthora* species, some other oomycetes like *Pythium* and *Hyaloperonospora* species, and even fungi, suggesting also a broad evolutionary conservation (Torres Ascurra, 2023). This offers the possibility of deploying PERU in additional crop species and improve resistance against a wider range of pathogens. Collectively, these examples highlight how PRRs can be strategically deployed for crop protection by leveraging their intrinsic ligand‐recognition specificities.

Beyond ligand recognition spectrum, the successful deployment of PRRs across species requires compatibility within the host machinery. Defense‐growth trade‐offs have been a major limitation in breeding resistance, especially in heterologous systems. A fine‐scale engineering driven by a better understanding of each structural module of the receptors and their functions promises to circumvent this limitation (Ngou et al., 2026a). A recent study has highlighted the importance of analyzing the structure of PRRs to engineer resistance efficiently. It has been shown that the C‐terminal (CT) domain of RLP23 is critical for compatibility and efficacy when transferred to other species (Yang, Steidele, Huang, et al., 2025). A chimeric RLP23 containing a tomato CT domain showed to provide stronger immune responses and greater disease resistance against bacterial, fungal, and oomycete pathogens than the original RLP23 when transferred to tomato, without compromising plant growth (Yang, Steidele, Huang, et al., 2025). It remains unknown whether a similar mechanism can be exploited to ensure the optimum heterologous expression of RKs.

PRRs diversification enables the recognition of PAMP variants that evolve to evade detection. For instance, *Vitis riparia* FLS2^XL^ and *Glycine max* FLS2b recognizes flg22 epitopes from *Agrobacterium tumefaciens* and *R. solanacearum*, respectively, that evade detection by canonical FLS2 receptors (Fürst et al., 2020; Wei et al., 2020). Ectopic expression of FLS2^XL^ in *Nicotiana tabacum*, and FLS2b in *S. lycopersicum* conferred enhanced resistance to *A. tumefaciens* and *R. solanacearum* (Fürst et al., 2020; Wei et al., 2020). In line with this, *Solanum* species bear diverse *PERU* alleles encoding PRRs with distinct ligand specificities (Torres Ascurra et al., 2023). Therefore, it is conceivable that certain PERU homologs may exhibit broader recognition spectra or detect Pep‐13 epitopes from adapted *P. infestans* strains that escape recognition by other PERU variants. Such receptors could represent valuable resources to generate broad‐spectrum and durable resistance against this destructive pathogen.

Engineering existing PRRs to broaden their ligand recognition represents a promising strategy to achieve durable resistance, particularly relevant for potato, given the scarcity of characterized PRRs. This approach was successfully demonstrated with FLS2, where selective reverse engineering of these receptors resulted in an expanded recognition of evading flg22 variants (Li, Jarquin Bolaños, et al., 2025; Zhang, Liu, et al., 2025), and with the SELECTIVE COLD SHOCK PROTEIN RECEPTOR (SCORE) that recognizes cold shock protein (CSP) peptides. SCORE was engineered to confer novel recognition of CSPs from pathogens like *Ralstonia*, *Xanthomonas*, *Candidatus* Liberibacter asiaticus, and root‐knot nematodes, ligands that are not recognized by the native SCORE (Ngou et al., 2025). These findings pave the way to engineer the potato immune receptors to expand their ligand recognition spectrum and ultimately improve disease resistance. However, a thorough analysis of natural polymorphism of their corresponding PAMPs is essential to guide those engineering efforts.

## NLRs—SECOND LINE OF DETECTION

Plant intracellular NLR immune receptors sense pathogen effector proteins that have been delivered into the plant cell (Dangl & Jones, 2001). NLRs are structurally diverse and generally classified by their N‐terminal domains into CC‐NLR (CNL), TIR‐NLR (TNL), and RPW8‐NLR (RNL) (Jones et al., 2016). In the resting state, NLRs are maintained in an autoinhibited conformation through complex intramolecular interactions across the N‐terminal, NB‐ARC, and LRR domains (Contreras, Pai, et al., 2023; Madhuprakash et al., 2024). Upon direct or indirect pathogen perception, these structural constraints are relieved, allowing the N‐terminal CC, TIR, or RPW8 domains to initiate downstream defense signaling (Duxbury et al., 2021). Although it was known that NLR‐triggered immunity leads to HR, the molecular mechanism remained largely unknown for many years (Jubic et al., 2019). This gap was resolved with the structural elucidation of the first NLR resistosome ZAR1 from Arabidopsis, which revealed that activated NLRs can oligomerize into wheel‐shaped complexes capable of associating with the plasma membrane (Wang et al., 2019). For many CNLs, activation leads to the exposure of a conserved N‐terminal MADA motif that is required for membrane targeting and is heavily implicated in forming the pore‐like structures necessary for HR execution (Adachi et al., 2019). Structural and functional analysis demonstrated that resistosomes form a Ca^2+^‐permeable cation channel, providing a direct mechanistic link between NLR activation and ion flux‐mediated cell death (Bi et al., 2021). Notably, the resistosome‐membrane association is not restricted to the plasma membrane; recent evidence demonstrates that resistosomes can dynamically engage with multiple single and double membrane organelles to effectively trigger NLR‐mediated immunity (Ibrahim et al., 2026). These findings shifted the view of NLRs from mere signaling adaptors to direct ion channel‐forming executors to trigger cell death. On the other hand, TNL activation follows a mechanistically distinct route in which after effector perception, the TIR domains oligomerize producing a signaling module that activates ENHANCED DISEASE SUSCEPTIBILITY 1 (EDS1)‐containing complexes and downstream helper RNLs, such as N REQUIREMENT GENE 1 (NRG1) or ACTIVATED DISEASE RESISTANCE 1 (ADR1) (Jacob et al., 2021). This process culminates in the production of ROS, ion fluxes, transcriptional reprogramming, and programmed cell death (Zhou & Zhang, 2020).

Numerous NLRs have been characterized in wild *Solanum* species and have been extensively studied in the context of resistance breeding against major potato diseases, such as late blight (Paluchowska et al., 2022). Most of these receptors have been cloned, predominantly belonging to the CNL class, and their corresponding recognized pathogen effectors have been identified (Table 1). Late blight resistance breeding efforts began with *Solanum demissum* as a donor of diverse *R* genes, such as *R1‐11* (Black et al., 1953; Paluchowska et al., 2022). Although these genes initially conferred high levels of race‐specific resistance, many were rapidly compromised due to the fast adaptive evolution of *P. infestans*. Resistance genes from *Solanum bulbocastanum*, such as *Rpi‐blb1* and *Rpi‐blb2*, initially showed broad‐spectrum resistance and promised more durability (Song et al., 2003; Van Der Vossen et al., 2003, 2005), but virulent strains are emerging too (Champouret et al., 2009; Li et al., 2012). Following *S. demissum* and *S. bulbocastanum*, a wide diversity of tuber‐bearing *Solanum* species has become an important source for *R* gene cloning and introgression breeding (Vleeshouwers, Finkers, et al., 2011; Vleeshouwers, Raffaele, et al., 2011).

### Helper and sensor NLRs

In contrast to the simplified binary view of the gene‐for‐gene model, NLRs frequently operate in pairs or networks utilizing distinct regulatory mechanisms. While some sensor NLRs function by negatively regulating and repressing the autoactivity of their paired helper, sensors and helpers also exist independently in the resting state, with pathogen perception prompting the activated sensor to trans‐activate its downstream helpers (Wu et al., 2020).

In Asterid plants, such as potato, CNL‐type helper NLRs, such as the NRC family form a complex genetic network. This robust network architecture, in which numerous diverse sensors funnel signals to a few central helpers, originated over 100 million years ago from a tightly linked sensor‐helper pair, preserved today as the NRC0 cluster (Goh et al., 2024; Sakai et al., 2024). In the tuber‐bearing *Solanum* section *Petota*, this ancient system underwent an explosive, asymmetric expansion (Wu et al., 2020). Uncoupled from physical linkage, sensors diversified in response to rapidly evolving pathogen effectors, while downstream helpers remained highly conserved, funneling diverse signals to a redundant core of helper nodes, primarily NRC2, NRC3, and NRC4 (Wu et al., 2020). These active defense pathways dynamically co‐evolved with pathogens, giving rise to distinct dependency relationships between sensors and helpers (Wu et al., 2020). For instance, the broad‐spectrum sensor Rpi‐blb2 relies strictly on NRC4 (Derevnina et al., 2021), whereas Rpi‐amr1 utilizes NRC2/NRC3 (Witek et al., 2021), and Rpi‐amr3 redundantly exploits all three (Lin et al., 2023). This circuitry operates alongside parallel networks, where RNL‐mediated TNL signaling (Jacob et al., 2021; Wu et al., 2019) runs alongside independent CC‐NLRs, such as Rpi‐vnt1, which possess both sensing and execution capacities (Wu et al., 2020).

Despite these parallel safeguards, reliance on centralized NRC hubs creates a potential bottleneck that pathogens can exploit (Derevnina et al., 2021). *Phytophthora infestans* deploys AVRcap1b to target the NRC execution machinery. Rather than associating with resting helpers, AVRcap1b employs a dual suppression mechanism: It bridges activated NbNRC2 to the vesicle‐trafficking protein NbTOL9a to hijack the host endosomal sorting complex required for transport (ESCRT) pathway (Madhuprakash et al., 2024), while independently binding SlNRC3 oligomerization intermediates to sterically block stepwise resistosome assembly and consequently suppress cell death (Seager et al., 2025). These examples reveal a new pathogen strategy that affects a whole immune complex assembly, highlighting vulnerabilities that can be exploited to build a more robust immune system.

### NLR transfer and engineering

Despite the identification of numerous NLRs in potato that confer resistance against *P. infestans*, durable resistance to this pathogen remains elusive. The identification of new *NLR* genes becomes challenging, emphasizing the need for new strategies to deploy these receptors. For instance, the stacking of three NLR genes (*RB*, *Rpi‐blb2*, and *Rpi‐vnt1‐1*) has shown promising results against *P. infestans* in field trials conducted in Africa (Ghislain et al., 2019), but for long‐term durability, new stacks would have to be deployed, as *P. infestans* will break down the resistance eventually. Conserved NRC hubs offer a great opportunity to overcome the restricted taxonomic functionality (RTF) observed when NLRs are nonfunctional in distantly related plants. Recent work demonstrated that the transfer of the *Rpi‐amr1* and *Rpi‐amr3* genes from *S. americanum* was able to confer effector recognition and trigger programmed cell death in soybean and Arabidopsis only when co‐expressed with their cognate NRC‐helper NLRs (Du et al., 2025). These findings indicate that NRC hubs can be used to deploy NLRs across evolutionary distances for disease resistance.

Advances in structural biology, functional genomics and artificial intelligence (AI)‐based structure prediction have importantly expanded the toolbox for NLR engineering. The cryo‐EM structures of activated CNL resistosomes, such as ZAR1, NRC2, and NRC4, in parallel with AI‐based structure prediction tools such as AlphaFold have enabled accurate modeling of NLR domains and NLR‐effector interfaces (Du et al., 2025; Liu et al., 2024; Madhuprakash et al., 2024; Wang et al., 2019). Beyond the conventional NLR architecture, some NLRs have acquired non‐canonical integrated domains (IDs) that function as decoys mimicking effector targets to trigger the plant immune response. Among the IDs, the heavy‐metal‐associated (HMA) domain is the most common among wild and cultivated potato accessions (Wang et al., 2026). A remarkable example is the recently described HMA‐containing resistant gene *Rpi‐brk1* from *Solanum burkartii*, which confers robust resistance to *P. infestans* (Wang et al., 2026). When the HMA domain from *Rpi‐brk1* was incorporated into the closely related *R1* gene from *S. demissum*, the chimeric protein gained the ability to recognize the effector AVRbrk1 while retaining its original AVR1 recognition. These ‘plug‐in’ IDs highlight the flexibility of NLRs and offer a promising strategy for engineering enhanced disease resistance in potato.

## COMPARING NLRs AND PRRs

### Convergence in signaling and dependency on helper networks

The traditional strict separation of PRR‐ and NLR‐mediated immunity has become increasingly blurred, as extensive molecular evidence indicates that these immune responses form a continuum rather than independent pathways (Thomma et al., 2011). This continuum is supported by observed convergence in downstream physiological responses, including the influx of Ca^2+^, ROS burst, activation of MAPK cascades, transcriptional reprogramming, and biosynthesis of defense phytohormones (Lu & Tsuda, 2021). The overlap extends to HR, traditionally considered a hallmark of NLR‐mediated immunity.

Notably, certain Solanaceous LRR‐RLPs are capable of triggering HR. For example, the LRR‐RLP Cf‐4 triggers HR upon detection of AVR4, in a NRC3‐dependent manner (Kourelis et al., 2022). The NRC network represents a key *Solanaceae*‐specific signaling module that may define a point of divergence from Arabidopsis, which lacks an equivalent helper network. Conservation of NRC3 across *Solanaceae* further *s*uggests a conserved link of cell surface and intracellular immune receptors in this family (Kourelis et al., 2022). The potato LRR‐RLP ELR also triggers HR upon detection of INF1 (Figure 1); however, it remains to be determined whether this response requires the NRC network.

More intriguingly, the LRR‐RK PERU triggers HR upon recognition of Pep‐13, representing a remarkable example of RK capable of inducing HR. Similarly to what has been reported for FLS2 (Wu et al., 2020), NRC2/3/4 are not required for PERU‐triggered HR or ethylene accumulation (Torres Ascurra et al., 2023). The downstream signaling pathway leading to HR therefore remains unresolved.

Another point of convergence for PRR‐ and NLR‐mediated signaling observed in model systems lies at the EDS1‐PAD4‐ADR1 node. Direct evidence in Arabidopsis demonstrates that RLP23 requires these signaling components (Pruitt et al., 2021; Torres Ascurra et al., 2023). In contrast, Solanaceous receptors show varying dependencies. While it is unknown if ELR follows the RLP23 model, the RK PERU, similar to FLS2, has been shown to prescind of this node, as it can trigger HR and ethylene accumulation independently of these components (Torres Ascurra et al., 2023). Together, these findings highlight that PRRs and NLRs can converge on downstream signaling hubs, but individual receptor families can differ in their specific signaling dependencies.

### Spatial compartmentalization

**Figure 2:**
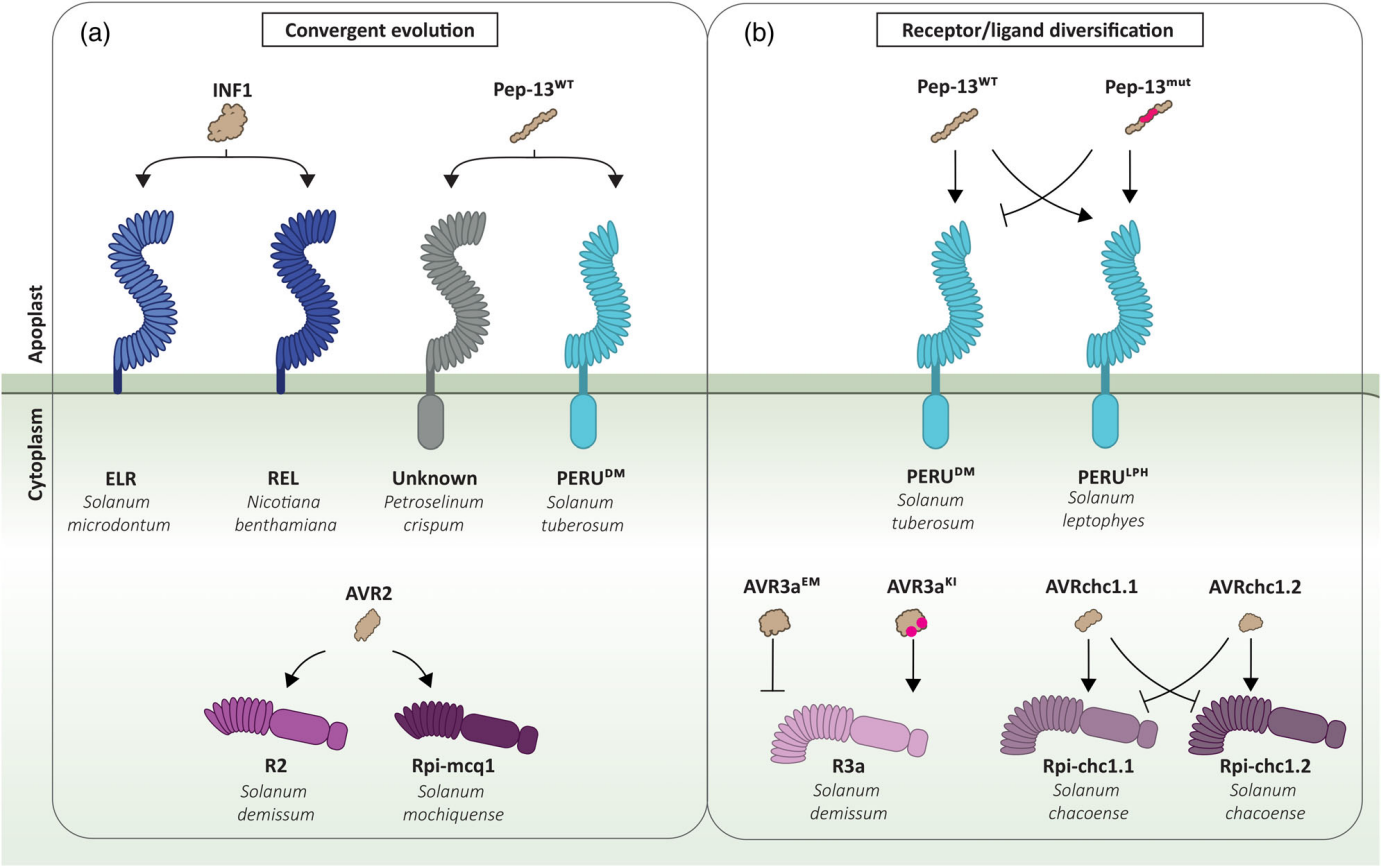
Coevolution of potato immune receptors and their ligands. (a) Convergent evolution: Independently evolved immune receptors recognize the same ligand. The unrelated PRRs REL (*Nicotiana benthamiana*) and ELR (*Solanum microdontum*) both recognize the *Phytophthora* elicitin INF1. Similarly, PERU^DM^ (*Solanum tuberosum*) and a still unidentified receptor in parsley (*Phytophthora* *crispum*) recognize the *Phytophthora* Pep‐13^WT^ ligand. Similarly, the NLR Rpi‐chc1.1 and Rpi‐chc1.2 specifically recognize *Phytophthora infestans* RXLR AVRchc1.1 and AVRchc1.2, respectively, but not vice versa. (b) Receptor–ligand coevolution: Immune receptors diversify to recognize distinct epitopes from PAMPs and effectors. PERU^DM^ only recognizes Pep‐13^WT^, while PERU^LPH^ also recognizes Pep‐13^mut^ that is not recognized by PERU^DM^. Similarly, the unrelated NLRs *R2* (Mexico) and *Rpi‐mcq1* (Peru) have independently coevolved to recognize AVR2 from *P. infestans*.

The principal distinction between NLRs and PRRs lies in their subcellular localization, with NLRs functioning intracellularly, whereas PRRs are positioned at the cell surface. This spatial separation underpins the division between intracellular and extracellular immunogenic patterns (Van Der Burgh & Joosten, 2019) (Figures 1 and 2). Intracellular NLRs detect effectors that localize to the cytoplasm or nucleus, such as the oomycete RXLR effectors that typically correspond to classical avirulence (AVR) genes. In contrast, cell surface‐localized PRRs perceive extracellular immunogenic patterns that include PAMPs and apoplastic effectors, such as small cysteine‐rich proteins, hydrolytic enzymes, and enzyme inhibitors (Saraiva et al., 2022).

### Evolutionary trajectories

In a similar way to the well‐characterized NLRs‐effectors arms race, recognition of PAMPs by PRRs imposes evolutionary pressure on pathogens to avoid recognition, leading to PAMP polymorphism that can escape detection, and in turn, drive the diversification of PRRs (Buscaill & Van Der Hoorn, 2021). Diversification of PRRs can occur through allelic variation of receptor orthologues as well as through convergent evolution of structurally unrelated receptors recognizing similar or identical PAMPs (Snoeck et al., 2025). For instance, the screening of wild and cultivated potato genotypes revealed that both wild and cultivated species bear diverse PERU alleles that encode PRRs with distinct ligand specificities. In particular, ligand binding and defense activation assays revealed that PERU^DM^ (from *S. tuberosum*) and PERU^LPH^ (from *Solanum leptophyes*) encode related LRR‐RK immune receptors that diversified in ligand specificities (Torres Ascurra et al., 2023) (Figure 2). Interestingly, 10 of 11 residues identified as being under positive selection are located on the concave ligand‐binding surface of the LRR structure of PERU, indicating that diversifying selection may have driven functional diversification of PERU receptors in wild potato populations.

Diversification of recognition specificity of potato NLRs, likely due to coevolution with *P. infestans*, has been shown for the *Rpi‐chc1* locus from *Solanum chacoense* (Monino‐Lopez et al., 2021). The alleles *Rpi‐chc1.1* and *Rpi‐chc1.2* recognize *P. infestans* RXLR PexRD12 and PexRD31, respectively (Figure 2). Despite high sequence similarity, polymorphisms in solvent‐exposed residues of the LRR domain determine their differential effector specificities. Detailed mutational analyses demonstrated that specific amino acid substitutions in the LRR region can shift recognition from PexRD12 to PexRD31, and vice versa. Importantly, the inactive allele *rpi‐chc_RH* from the *S. tuberosum* RH89‐039‐16 does not confer PexRD12/31 recognition, resulting in susceptibility to *P. infestans*. Targeted LRR domain exchanges with the active *Rpi‐chc1.1* and *Rpi‐chc1.2* alleles were sufficient to restore effector recognition, converting the inactive allele into a receptor capable of recognizing either PexRD12 or PexRD31 effectors (Monino‐Lopez et al., 2021).

Beyond lineage‐specific diversification and arms race dynamics, also convergent evolution has shaped immune receptor repertoires. ELR from potato and the unrelated REL receptor in Nicotiana evolved independently to recognize elicitins of *Phytophthora* (Chen et al., 2023; Du et al., 2015) and the potato receptor PERU and a distinct yet unknown receptor in parsley perceived the *Phytophthora* epitope Pep‐13 (Figure 2). NLRs of the *R2* family in Mexican *Solanum* species as well as the genetically unrelated *Rpi‐mcq1* family from Peru independently coevolved with *AVR2* from *P. infestans* (Aguilera‐Galvez, 2019; Aguilera‐Galvez et al., 2018; Jones et al., 2014). These cases illustrate how independent receptor lineages can converge on recognition of the same pathogen‐derived molecules, while diversification of PRRs and NLRs enables plants to adapt to rapidly evolving pathogens.

**Figure 3:**
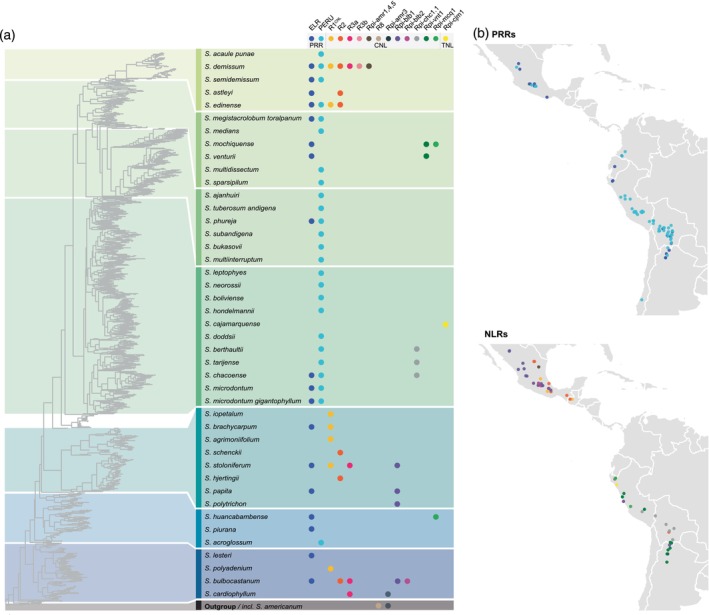
Phylogenetic and geographic classification of *Solanum* species and their immune receptors involved in recognition of *Phytophthora infestans*. (a) Distribution of functional potato *PRR* and *NLR* genes across *Solanum* accessions arranged according to their phylogenetic relationships based on Jacobs et al. (2008). Clade shading indicates major phylogenetic groups and dots mark the presence of *PRR* or *NLR* genes identified through effector/ligand recognition assays or disease tests (Aguilera‐Galvez, 2019; Du, 2014; Rietman, 2011; Torres Ascurra et al., 2023) with colors corresponding to specific gene identities. *NLR* genes occur in a limited number of clades, whereas *PRRs* are distributed more broadly across the phylogeny. (b) Geographic origin of *Solanum* species carrying *PRR* (top) or *NLR* (bottom) genes conferring responsiveness to *P. infestans*. Circles indicate collection sites of accessions harboring functional genes, with colors representing *PRR* or *NLR* gene identity. CNL, coiled‐coil (CC) NLR; NLR, nucleotide‐binding leucine‐rich repeat; PRRs, pattern recognition receptors; TNL, toll/interleukine‐1 receptor‐nucleotide‐binding leucine‐rich repeat.

Potato immune receptors conferring resistance to *P. infestans* display distinct geographic and phylogenetic distribution patterns that reflect their evolutionary history within *Solanum*. Core PRRs such as FLS2 are highly conserved in potato and across angiosperms, indicating an ancient origin (Torres Ascurra et al., 2023; Zhang, Liu, et al., 2025; Zipfel et al., 2004). Similarly, ELR is broadly conserved across multiple clades within *Solanum* section *Petota* (Du, 2014) (Figure 3), supporting the view that key PRRs constitute stable components of basal immunity. Although exceptions exist, such as the genetically diverse PERU receptor, which appears enriched in younger Andean lineages.

Whereas these PRRs generally tend to display deep evolutionary conservation and wide phylogenetic distribution, sensor *NLRs* (*Rpi* genes) tend to exhibit more restricted phylogenetic and geographic distributions, consistent with localized coevolutionary dynamics between *P. infestans* and its *Solanum* hosts (Figure 3). Most well‐characterized NLRs show strong regional associations; for example, *Rpi‐blb1, Rpi‐blb2, Rpi‐blb3*, and *R3a* are broadly maintained in Mexican *Solanum* species that thrive in Toluca Valley, that is a recognized center of diversity for *P. infestans* (Lokossou et al., 2010), and *Rpi‐mcq1, Rpi‐vnt1*, and *Rpi‐chc1.1* are largely confined to Peru, Argentina, and Bolivia, respectively, supporting the idea of relatively recent, spatially confined evolutionary origins (Table 1; Figure 3). Some *Rpi‐amr* genes from *S. americanum*, which predates the evolution of the tuber‐bearing *Petota*, have been retained across later‐diverging species, demonstrating that some NLRs can still exhibit deep evolutionary persistence (Lin et al., 2023; Wang et al., 2026). In contrast to these sensor NLRs that are typically considered type I, fast evolving receptors, the helper NLRs are more conserved and belong to the type II classification (Kuang et al., 2004; Wang et al., 2026). Collectively, these observations suggest that potato immune receptor evolution follows a continuum rather than a strict dichotomy, with PRRs generally representing evolutionarily ancient and broadly retained components, while sensor NLRs more often reflect geographically structured and rapidly diversifying lineages, albeit with notable exceptions in both classes.

### Response intensity and contribution to resistance

Since the concept of the zigzag model emerged, NLR‐mediated immunity has generally been viewed as an amplified version of PRR‐mediated immunity that often surpasses a threshold for induction of HR (Jones & Dangl, 2006). Indeed, most *NLRs* (*Rpi* genes) derived from wild *Solanum* species and introduced into potato cultivars mount an HR that leads to complete resistance to *P. infestans*. In contrast, ELR and PERU enhance resistance to *P. infestans* at a quantitative level (Du et al., 2015; Torres Ascurra et al., 2023). Similarly, expression of RLP23, a PRR from Arabidopsis, in potato led to a measurable, albeit modest increase in resistance (Albert et al., 2015; Yang, Steidele, Löffelhardt, et al., 2025).

As mentioned in previous paragraphs, most PRRs activate immune responses without inducing HR, whereas PERU and ELR trigger HR upon recognition of Pep‐13 and the INF1 elicitin, respectively (Torres Ascurra et al., 2023; Weralupitiya et al., 2024). Remarkably, neither PERU nor ELR are able to mount HR during *P. infestans* infection. This may reflect suppression of PRR‐mediated immunity by effectors, and if so, stacking PRRs with NLRs that recognize the corresponding suppressive effector could potentially result in a stronger and durable defense response (Albert et al., 2015; Du et al., 2015; Torres Ascurra et al., 2023; Yang, Steidele, Huang, et al., 2025).

Although PRRs represent promising targets for engineering durable resistance that may outlast the rapidly overcome resistance conferred by many NLRs, their effective deployment against pathogens such as *P. infestans* in agriculture may require strategies to significantly enhance PRR‐mediated immunity (Box 1). Despite the apparent contrast between the level of resistance provided by NLRs and PRRs in the potato—*P. infestans* interaction, this distinction should not be considered as absolute. Studies in diverse other pathosystems have demonstrated that both receptor classes can mediate defense responses ranging from quantitative to robust complete resistance, depending on each specific interaction (Contreras, Lüdke, et al., 2023; Yuan, Ngou, et al., 2021).

Box 1 **General summary message**

- Wild potato species harbor an extensive and dynamic repertoire of immune receptors.
- PRRs generally represent evolutionarily conserved components of basal immunity.
- NLRs generally exhibit geographically structured diversification driven by local pathogen pressure.
- Unlike typical PRRs, the PERU family exhibits diversification in natural populations, likely shaped by local pathogen pressure as well.
- Rational bioengineering of immune receptors based on integrating evolutionary insights and AI‐guided design offers new opportunities for generating durable resistance.

## FUTURE PERSPECTIVES

### Precision breeding era

Recently initiated diploid hybrid potato breeding strategies based on inbred lines enable true seed propagation and simplify genetic analysis (Lindhout et al., 2011; Stokstad, 2019) and offer opportunities to accelerate resistance breeding compared to the conventional breeding of tetraploid potato. Gene pyramiding against the reemerging late blight, plus introducing multiple *R* genes to protect against the many diseases that threaten the potato crop, becomes more feasible. The recently reported interfamily transfer of *Solanaceae* sensor and helper NLRs that break restricted taxonomic functionality unlocks the deployment of NLRs for other crops' protection (Du et al., 2025) and likely from other crops to potato. In parallel, the development of new genomic technologies (NGTs) such as cisgenesis and targeted genome editing offers additional opportunities to introduce or stack resistance genes with high precision. Ideally, this should be complemented with monitoring the pathogen population for genetic variation of effectors and integrated crop management tools. Ongoing regulatory discussions within the European Union, including proposals by the European Commission and deliberations in the European Parliament, may facilitate broader deployment of educated NGT approaches in potato breeding. Nevertheless, continuous introduction of new immune receptors remains essential to keep up in the arms race with pathogens (Box 2).

Box 2 **Remaining open questions**

- Can RK be structurally engineered to ensure interspecies compatibility, with the same effectiveness as RLPs?
- How can PRR‐mediated quantitative resistance be enhanced to agronomically meaningful levels?
- How should next‐generation technologies (NGTs) be integrated into resistance deployment strategies?
- Can elucidating the downstream signaling networks underlying the hypersensitive response triggered by potato PRRs lead to rational stacking of immune receptors?
- Do bioengineered potato immune receptors with improved recognition lead to broader and/or stronger disease resistance?

### Evolutionary‐guided receptor engineering

The emerging EvoMPMI field provides insights into how immune receptors and pathogen ligands have evolved to acquire their current functions. A highly diverse set of NLR receptors have been cloned from wild *Solanum* species and PRR are being uncovered as well, along with the matching effectors and PAMPs, respectively. This outstanding genetic resource allows evolutionary studies, and distinguishing ancestral and derived sequences can provide crucial insights to place functional molecular changes into a broader mechanistic framework (Box 2).

### AI‐driven receptor engineering

The utilization of AI methods to predict protein–ligand interactions can expedite the identification of critical residues within the receptor–ligand binding sites (Ibrahim et al., 2023; Li, Jarquin Bolaños, et al., 2025; Snoeck et al., 2024), and this can be further extended to tweaking components downstream of the receptor. Elegant receptor bioengineering studies for the model FLS2 (Li, Jarquin Bolaños, et al., 2025; Zhang, Liu, et al., 2025) have proven that broader recognition spectra can be achieved. Potato PRRs show a highly dynamic evolution compared to canonical PRRs, providing huge natural resources to be leveraged by AI. The concept of AI‐based bioengineering of immune receptors can be further extended to candidate virulence targets and signaling components, to achieve the best possible scenario for elevating PRR/NLR‐mediated disease resistance (Box 2).

## CONFLICT OF INTEREST

None of the authors have a conflict of interest to disclose.

## Data Availability

Data sharing not applicable to this article as no datasets were generated or analyzed during the current study.
